# Effectiveness of a multi-component facility-based intervention on HIV-related infant and maternal outcomes: results from the IMPROVE clustered randomized study

**DOI:** 10.3389/fmed.2025.1521564

**Published:** 2025-04-07

**Authors:** Lauren Greenberg, Vincent J. Tukei, Heather J. Hoffman, Ramatlapeng Thabelo, Tsietso Mots’oane, Matsepeli Nchephe, Mammatli Chabela, Matseliso Masitha, Majoalane Mokone, Amy Knowlton, Shannon Viana, Lynne Mofenson, Appolinaire Tiam, Laura Guay

**Affiliations:** ^1^Elizabeth Glaser Pediatric AIDS Foundation, Washington, DC, United States; ^2^Elizabeth Glaser Pediatric AIDS Foundation, Maseru, Lesotho; ^3^Department of Biostatistics and Bioinformatics, Milken Institute School of Public Health, George Washington University, Washington, DC, United States; ^4^Ministry of Health, Maseru, Lesotho; ^5^Johns Hopkins Department of Health Behavior and Society, Johns Hopkins Bloomberg School of Public Health, Baltimore, MD, United States; ^6^Department of Epidemiology, Milken Institute School of Public Health, George Washington University, Washington, DC, United States

**Keywords:** HIV, prevention of vertical HIV transmission, maternal-child health, multidisciplinary teams, Lesotho

## Abstract

**Introduction:**

Even in the context of widespread access to prevention of vertical HIV transmission (PVT) services, health system challenges compromise health outcomes for women living with HIV and their children. The “Integrated Management Team to Improve Maternal-Child Outcomes” (IMPROVE) study measured the effect of a package of facility-based interventions on PVT and maternal and child health (MCH) outcomes in Lesotho.

**Methods:**

This cluster-randomized study included six facilities randomized to the standard-of-care and six to the IMPROVE intervention. The intervention included multidisciplinary teams of health care and community workers providing MCH support, training in patient-centered care, and additional home support. Pregnant women with and without HIV were enrolled at their first antenatal visit and followed through 12–24 months postpartum with their infants. Data were collected through participant interviews and routine medical record abstraction. Primary outcomes included viral suppression and adherence to antiretroviral therapy (ART) for women with HIV and repeat HIV testing for women without HIV. Analysis utilized generalized estimating equations (GEE) adjusted for intra-site correlation.

**Results:**

Between July 2016 and February 2017, 614 pregnant women with HIV and 390 without HIV were enrolled. At 12 months postpartum, over 90% of women with HIV with viral load (VL) testing had a VL < 1,000 copies/mL; the intervention arm had a trend toward higher proportion with undetectable VL (< 50 copies/mL) compared to the control arm [83% versus 72%, OR 1.9 (95% CI 0.86–4.14)]. Women with HIV in the intervention arm had significantly higher odds of consistent adherence to ART [OR 1.81 (95% CI 1.03–3.18)], and women without HIV in the intervention arm had significantly higher odds of being re-tested for HIV prior to delivery [OR 1.95 (95% CI 1.23–3.08)].

**Conclusion:**

Sites that implemented the IMPROVE intervention documented better PVT and MCH outcomes than sites implementing standard-of-care. This package of facility-based interventions is a promising and easily scalable model for improving coordination, quality, and uptake of services within the existing health system.

## Introduction

Despite significant global progress toward reducing vertical HIV transmission, the rate of decline in the number of children with newly acquired HIV infection has slowed in recent years; UNAIDS estimates that approximately 120,000 children were newly infected with HIV in 2023 (1). Barriers to service uptake, poor maternal retention and antiretroviral treatment (ART) adherence, and health system challenges continue to contribute to vertical transmission and to compromise health outcomes for mothers living with HIV and their children (1–7).

Failures across all four prongs of the World Health Organization (WHO) prevention of vertical HIV transmission (PVT) strategy contribute to ongoing vertical transmission. Focused counseling and support for primary prevention among pregnant women without HIV (PVT prong 1) are limited in PVT programs (8). Coupled with low rates of retesting, high HIV sero-incidence among pregnant and postpartum women in Sub-Saharan Africa remain barriers to elimination of vertical transmission; UNAIDS estimates that incident infections during pregnancy or breastfeeding account for 32% of new vertical infections in eastern and southern Africa (1, 9–12). Efforts to reduce unplanned pregnancies (PVT prong 2) and increase access to family planning are often lacking (13–15). While access to PVT services and ART for pregnant women with HIV has improved (PVT prong 3), rates of retention in care and ART adherence have been found to be lower for women in PVT programs than in non-pregnant persons with HIV in some settings. Approximately 27% of new vertical infections in eastern and southern Africa in 2023 are attributed to women with HIV who interrupt ART during pregnancy or breastfeeding (1, 2). Loss to follow-up (LTFU) and poor ART adherence are particularly high within the first 3 months after ART initiation and during the postpartum period (2–6). Stigma (including from healthcare providers), lack of disclosure, and limited family and community support (PVT prong 4) also contribute to poor retention (2, 7, 8, 16, 17).

Health system barriers also affect PVT uptake and retention: poor communication and coordination between PVT services and other health services and between different cadres of providers; negative attitudes of health care workers (HCW); and inadequate counseling and support of women with HIV at ART initiation and across the PVT cascade (1, 2, 4, 7, 18, 19). Multiple strategies shown to be effective individually in study settings to improve PVT program support and retention, such as the use of peer counselors, community health workers (CHWs), and support groups, have now been widely implemented in maternal and child health (MCH) services, yet LTFU and vertical transmission remain high in many African settings (18, 20–24). Use of multidisciplinary teams (MDT) to ensure patients receive a full range of care and support services has been successful for the management of complex non-HIV conditions (25–28). In resource-limiting settings, MDT may also facilitate task shifting and coordination among health cadres (29). Patient-centered care practices are also associated with better HIV treatment uptake, adherence, and viral suppression (30–32).

Lesotho has one of the highest HIV burdens globally, with an estimated HIV prevalence of 25.9% among pregnant women (33). Lesotho began offering lifelong ART for pregnant and breastfeeding women with HIV in 2013 and transitioned to the “Test and Treat” model in 2016; in 2023, UNAIDS estimated PVT coverage in women with HIV in Lesotho at 93% (34). With an estimated sero-incidence of 2.61 and 1.36 per 100 person-years in Lesotho during pregnancy and postpartum, respectively (35), elimination of vertical transmission requires a comprehensive approach to service delivery to both women with and without HIV during antenatal and postnatal care. We evaluated implementation of a multi-component facility-based intervention (IMPROVE) designed to combine known effective strategies in a coordinated approach for PVT and maternal-child health outcomes in Lesotho.

## Materials and methods

### Design and setting

We conducted a cluster-randomized prospective cohort study to evaluate the “Integrated Management Team to Improve Maternal-Child Outcomes” (IMPROVE) intervention in 12 rural and urban health facilities in Maseru District Lesotho from July 2016 to July 2019. Health facilities in the district serving between 150 and 900 pregnant women annually were categorized by size, type of facility (hospital or health center), hospital catchment areas, and type of support (government or Christian Health Association of Lesotho (CHAL)]. From this list, the study team selected both of two main hospitals and created two clusters by grouping each hospital with another five mid- to high-volume facilities within their referral area to avoid potential cross-contamination if a health facility and the hospital that received that facility’s referrals were randomly allocated to different arms. These five facilities were selected to ensure even distribution across size, facility type, and type of support. One cluster was randomly assigned to receive the IMPROVE intervention, and the other was designated as the standard-of-care (control) arm.

### Standard of care MCH and PVT services

All study facilities provided free MCH and PVT services, including HIV testing and ART. Women newly identified with HIV received same-day ART initiation. Guidelines for retesting women who initially tested negative for HIV included retesting at 36 weeks gestation and yearly thereafter during breastfeeding. Postnatal care was provided to women through 14 weeks after delivery. Women with HIV received HIV follow-up care every 1–3 months with yearly viral load (VL) testing. Infant postnatal care included 8 visits within the first 18 months of life. HIV testing for infants born to mothers with HIV was done at 6 weeks, 14 weeks, 9 months, and 18 months. In all facilities, clinical staff were supported by a cadre of peer counselors and CHW. In the event of a missed clinic appointment, women with HIV- like all other clients with HIV- were referred to dedicated facility staff and would receive an initial phone call 1 week after the missed visit. If the woman could not be reached or did not return for a visit, facility staff would enlist the appropriate community partner to conduct a home visit. Procedures for women without HIV were more variable at the time of this study; women may or may not have received a phone call or home visit.

### The IMPROVE intervention

The IMPROVE intervention included three key interventions: (i) formation of multidisciplinary, integrated management teams of facility- and community-based health care and lay workers, (ii) Joint Positive Health Dignity, and Prevention (PHDP)-focused counseling, skills-building training, and job aids to strengthen the MDT’s ability to provide consistent messages across all staff cadres (nurses, CHW, peers) and provide a patient-centered approach to identify solutions to barriers to care; and (iii) increased early community-based counseling and support to minimize early LTFU (including at least one additional home or community outreach visit within 2 weeks of the first ANC visit). At each intervention site, the study team supported the creation of a MDT, trained the MDT team in problem-solving techniques, and facilitated a group mapping exercise to optimize the patient flow within the facility and to identify gaps and potential strategies for ensuring patient follow-up and continuity of care from facility to the community. Thereafter, each MDT organized and ran their own meetings with documentation of attendance, minutes, and action plans. Further details on the IMPROVE intervention and the specific strategies adopted by each intervention site are described in Beres et al., 2024 (36).

### Study population

We enrolled pregnant women with and without HIV attending their first antenatal care (ANC) visit at a study site between July 2016 and February 2017. Women were eligible if they were residing in the catchment area for a study facility with no plans to relocate after delivery and provided written informed consent. Study nurses screened all pregnant women with HIV and a randomly selected subset of pregnant women without HIV (∼ one woman without HIV/week/facility) for study eligibility and enrolment. Both routine services and study activities were targeted toward individual women; while women could choose to bring a partner or other support person to their care visits or disclose their participation in the study, there was no formal mechanism for engagement with individuals other than the participant.

### Study procedures

Study visits were scheduled every 3 months until 12–24 months after delivery for women and their infants. Participants were interviewed and maternal and child clinical and laboratory data were abstracted from medical records. Data were entered directly into a cloud-based electronic database on the CliniOps platform (37). Dried blood spot specimens for VL were collected from women with HIV at 12 and 24 months postpartum. Specimens were transported to the National Reference Lab in Maseru and stored in the freezer until batched and sent to the National Institute for Communicable Diseases in Johannesburg, South Africa for testing using Roche COBAS Amplicor/Taqman assay.

### Outcomes

Primary outcome measures included viral suppression and ART adherence for women with HIV, repeat HIV testing for women initially testing negative for HIV, and other selected MCH behaviors and outcomes for all women and children. VL was defined as undetectable if < 50 copies/mL and suppressed if < 1,000 copies/mL. Adherence was defined as taking ≥ 95% of ART doses (assessed by facility staff through pill counts at each visit and recorded in the patient’s file). Data on adherence were reviewed and summarized as either “consistently adherent” (participant adherent at each visit where documented), or “not consistently adherent” (at least one visit with documented sub-optimal adherence). “Modern contraception” was defined as use of any of the following to prevent pregnancy: barrier methods (condom, diaphragm, etc.), pill, injection, implant, IUD, or female or male sterilization. Modern contraception use was categorized as “consistent use” (reported at each post-partum visit 14 weeks or later), “mixed use” (use at some but not all visits), or “consistent non-use” (no use of modern contraception at any postpartum study visit at 14 weeks or later). Patient satisfaction was assessed after each routine care visit; at the end of the visit, clinic staff would direct the study participant to a separate area within the facility to meet with the study nurse and answer a series of questions, including about their experiences receiving care on that day.

### Statistical analyses

To summarize key indicators, we calculated descriptive statistics (frequency tables, mean/median/range). Bivariate tests of the association were performed using the Rao-Scott chi-square test, adjusting for clustering by site. Generalized estimating equations (GEE) using the binomial distribution, a logit, cumulative logit or generalized logit link function, and a compound symmetry working correlation structure were used to evaluate the relationship between study arm and key outcomes. We present odds ratios (ORs) adjusted for baseline characteristics identified *a priori* (maternal age, marital status, and education); models of adherence and use of modern contraception are also adjusted for the number of study visits for each participant, and the model of number of ANC visits was also adjusted for estimated weeks gestation at first ANC visit. In all models including both women with and without HIV, we tested for interaction between HIV status and study arm and present stratified results if the interaction was significant. No imputation or other adjustments were made for missing data; for outcomes incorporating data from multiple visits or time points, outcomes were derived using only data from visits that were completed as it was not possible to reliably impute values for missed visits. Analyses were conducted using SAS version 9.4 (SAS Institute Inc., Cary, NC, United States).

### Ethical considerations

The study protocol was approved by the Lesotho National Health Research Ethics Committee (NHREC), the George Washington University Institutional Review Board (GW-IRB) and the Population Council IRB. All participants provided written informed consent. The study is registered under ClinicalTrials.gov Identifier: NCT04598958.

## Results

**FIGURE 1:**
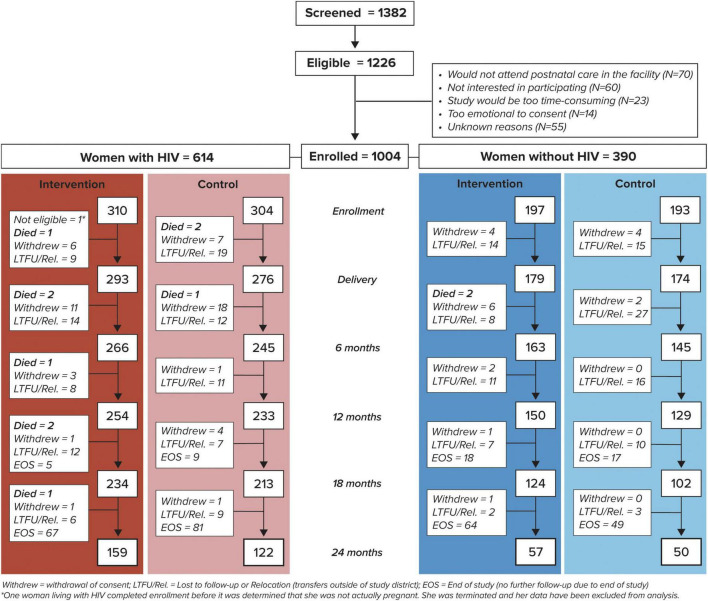
Study enrollment and follow-up of women by human immunodeficiency virus (HIV) status and study arm.

A total of 1004 pregnant women were enrolled: 310 pregnant women with HIV (one woman was not pregnant and was excluded from the analysis) and 197 pregnant women without HIV at intervention sites, and 304 pregnant women with HIV and 193 pregnant women without HIV at control sites (Figure 1). Of 613 eligible pregnant women with HIV enrolled, 80.6% had ≥ 12 months of study follow-up after delivery or had died (83.5% vs. 77.6% in the intervention and control arms, respectively). Among 390 pregnant women without HIV, 72.1% remained in active follow-up at 12 months or had died (77.2% vs. 66.8% in the intervention and control arms, respectively) (Figure 1). After adjustment, there was a non-significant trend for improved odds of retention through 12 months postpartum for women in the intervention compared to control arm (79.7% vs 72.8%, respectively, adjusted odds ratio (aOR) 1.5, 95% CI 0.95–2.44). Due to an unexpectedly long time to complete enrollment, the study ended prior to all participants reaching the 24 months endpoint in order to ensure that adequate time and resources were available for data cleaning and analysis.

### Baseline characteristics

Among pregnant women with HIV, the study arms were comparable in all baseline characteristics, with median age 28 years and median estimated gestational age at first ANC visit 20 weeks (Table 1). The majority (72.0%) of participants with HIV already knew their status at enrollment. For pregnant women without HIV, the median age was 23 years; participants without HIV in the intervention arm were significantly older, more likely to be married or living with partner, and had more previous pregnancies, but other characteristics were comparable.

**TABLE 1:** Maternal characteristics at enrollment (first antenatal visit).

	Pregnant women without HIV			Pregnant women with HIV		
**Maternal characteristics at enrollment (first ANC visit)**	**Control (*N* = 193)**	**Intervention (*N* = 197)**	**Total (*N* = 390)**	**Control (*N* = 304)**	**Intervention (*N* = 309)**	**Total (*N* = 613)**
**Maternal age at enrollment (years)**						
Median (Min-max)	22 (14–42)	24.(16–39)	23 (14–42)	28 (16–48)	28.5 (16–44)	28 (16–48)
< 19	**33 (17.1)**	**15 (7.6)**	**48**	15 (4.9)	9 (2.9)	24
19–24	102 (52.9)	96 (48.7)	198	80 (26.3)	73 (23.7)	153
25**+**	58 (30.1)	86 (43.7)	144	209 (68.8)	226 (73.4)	435
Missing					1	1
**Current marital status**						
Married/living with a partner	**134 (69.4)**	**158 (80.2)**	**292**	233 (76.6)	247 (80.2)	480
Not married/living with a partner	59 (30.6)	39 (19.8)	98	71 (23.4)	61 (19.8)	132
Missing					1	1
**Highest level of education**						
Primary education or less	38 (19.7)	51 (25.9)	89	112 (37.0)	109 (35.4)	221
Any secondary education	155 (80.3)	146 (74.1)	301	191 (63.0)	199 (64.6)	390
Missing				1	1	2
**Gravidity**						
1	**107 (55.4)**	**75 (38.9)**	**182**	73 (24.3)	56 (18.4)	129
2	58 (30.2)	68 (35.2)	126	108 (36.0)	121 (39.8)	229
3	19 (9.9)	34 (17.6)	53	64 (21.3)	71 (23.4)	135
4 or more	8 (4.2)	16 (8.3)	24	55 (18.3)	56 (18.4)	111
Missing	1	4	5	4	5	9
**Partner HIV Status**						
Unknown (including Not Tested)	106 (55.2)	97 (49.2)	203	82 (27.1)	71 (23.1)	153
HIV-positive	6 (3.1)	6 (3.1)	12	104 (34.3)	104 (33.8)	208
HIV-negative	80 (41.7)	94 (47.7)	174	117 (38.6)	133 (43.2)	250
Missing	1		1	1	1	2
**Estimated gestational age, first ANC visit (weeks)**						
N	191	193	384	293	299	592
Median (min–max)	22 (5–39)	21 (5–39)	22 (5–39)	21 (5–38)	20 (5–40)	20 (5–40)
Missing	2	4	6	11	10	21
Newly diagnosed with HIV	–	–	–			
Yes				86 (28.4)	85 (27.6)	171
No				217 (71.6)	223 (72.4)	440
Missing				1	1	2

Bold values indicate differences by study arm significant at *P* < 0.05. ANC, antenatal care; Min-max, minimum/maximum.

### Selected MCH outcomes

MCH outcomes were analyzed in the overall population (including women with and without HIV) (Table 2); results are presented separately by HIV status if there was a significant interaction between study arm and HIV status in adjusted analysis. Overall, women in the intervention arm had higher odds of more ANC visits compared to women in the control group, though the difference was not statistically significant in adjusted analysis (aOR: 1.55, 95% CI: 0.86–2.77). For example, 60.3% of women in the intervention arm completed the recommended ≥ 4 ANC visits compared to 48.0% of women in the control arm. More than 88% of women in each group delivered in a facility, with no significant difference by study arm. A significantly higher proportion of women in the intervention arm reported consistent postpartum use of modern contraception than in the control arm (aOR: 1.62, 95% CI: 1.05–2.5).

**TABLE 2:** Maternal and child health outcomes by study arm.

	Control	Intervention	Adjusted model (number patients)	aOR (95% CI)	Wald *p*-value
**ANC visits (number)**					
1	46 (10.6)	34 (7.4)	874	1.55 (0.86–2.77)^#^	0.143
2	69 (15.9)	58 (12.6)			
3	111 (25.5)	90 (19.6)			
4 or more	209 (48.0)	277 (60.3)			
Missing	4	2			
**Facility delivery**					
Yes	388 (88.4)	418 (91.1)	893	1.43 (0.70–2.92)	0.330
No	51 (11.6)	41 (8.9)			
**Postpartum (14+ weeks) use of modern contraception**					
Consistent use	133 (31.4)	199 (44.2)	872	1.62 (1.05–2.5)^##^	0.028
Mixed use	234 (55.3)	216 (48.0)			
Consistent non-use	56 (13.2)	35 (7.8)			
**Ever disclosed to partner (disclosure reported at any study visit, women with HIV only)**					
Yes	223 (73.4)	234 (75.7)	997	1.14 (0.48–2.72)	0.175
No	81 (26.6)	75 (24.2)			
**Ever disclosed to partner (disclosure reported at any study visit, women without HIV only)**					
Yes	108 (56.8)	146 (74.5)		2.05 (0.87–4.83)	
No	82 (43.2)	50 (25.5)			
Missing	3	1			

^#^Model also adjusted for estimated gestation at enrollment.

^##^Model also adjusted for number of postpartum study visits with data on contraception. ANC, antenatal care; aOR, adjusted odds ratio.

### Selected HIV outcomes

All pregnant women with HIV and a delivery outcome received ART, with approximately half on ART prior to pregnancy and the remainder initiating ART during pregnancy. ART adherence was measured by pill count as part of each routine HIV care visit. Of 582 women with HIV with data on ART adherence, the odds of consistent adherence were significantly higher among women in the intervention arm after adjusting adherence data for selected maternal characteristics (as noted earlier under “Materials and methods”) and number of study visits (aOR 1.81, 95% CI: 1.03–3.18) (Table 3).

**TABLE 3:** Human immunodeficiency virus (HIV)-related Outcomes between participants by study arm.

Selected outcomes	Control	Intervention	Total	*P*-value	Adjusted model (number)	Adjusted aOR (95% CI)
**Women with HIV only**						
**Consistent adherence**						
Yes	188 (65.5)	226 (76.6)	414	0.003	581	1.81 (1.03–3.18)
No	99 (34.5)	69 (23.4)	168			
**VL suppressed at 12 months**						
Suppressed (< 1,000 c/mL)	158 (90.3)	166 (94.9)	324	0.081	349	1.87 (0.90–3.91)
Unsuppressed ≥ 1,000 c/mL)	17 (9.7)	9 (5.1)	26			
**VL undetectable at 12 months**						
Undetectable (< 50 c/mL)	126 (72.0)	146 (83.4)	272	0.037	349	1.88 (0.86–4.14)
Detectable (≥ 50 c/mL)	49 (28.0)	29 (16.6)	78			
**VL suppressed at 24 months**						
Suppressed (VL < 1,000 c/mL)	100 (91.7)	129 (92.1)	229	0.841	249	0.77 (0.42–1.43)
Unsuppressed (≥ 1,000 c/mL)	9 (8.3)	11 (7.9)	20			
**VL undetectable at 24 months**						
Undetectable (< 50 c/mL)	89 (81.7)	107 (76.4)	196	0.179	249	0.68 (0.45–1.06)
Detectable (≥ 50 c/mL)	20 (18.4)	33 (23.6)	53			
**Women without HIV only**						
**HIV retesting prior to delivery**						
Yes	96 (63.6)	129 (77.3)	225	< 0.001	318	1.95 (1.23–3.08)
No	55 (36.4)	38 (22.8)	93			
**HIV retesting between delivery and 12 months**						
Yes	88 (69.8)	112 (75.2)	200	0.530	275	1.29 (0.47–3.51)
No	38 (30.2)	37 (24.8)	75			

aOR, adjusted odds ratio; c/ml, copies/mL; VL, viral load.

VL results were available for 350/487 (72%) women with HIV in the study at 12 months postpartum. The rates of suppression and undetectability were high overall (92.6% and 77.7%, respectively). Although women with HIV in the intervention compared to control arm had a higher proportion with suppressed VL (94.9% vs 90.3%) and undetectable VL (83.4% vs 72.0%), this did not reach statistical significance in the adjusted models (Table 3). Among 249 participants with VL results at 24 months, the proportion of women with viral suppression remained over 90%, with no difference in suppression or undetectable VL between arms.

Women without HIV in the intervention arm had significantly higher odds of retesting for HIV between enrollment and delivery (77.3% compared to 63.6% in the control arm; aOR 1.95, 95% CI: 1.23–3.08) (Table 3). The majority of women without HIV in both study arms received at least one repeat HIV test between delivery and 12 months postpartum. While the odds of postpartum retesting were higher among women in the intervention arm (75.2% vs. 69.8% in the control arm), this difference was not statistically significant.

Four women without HIV seroconverted to HIV during the study - three women in the control arm and one woman in the intervention arm (Table 4). All women who seroconverted were initiated on ART [efavirenz (EFV)/lamivudine (3TC)/tenofovir disoproxil fumarate (TDF), standard of care at the time]. One of their infants was identified as having acquired HIV at the same visit that seroconversion was documented.

**TABLE 4:** Characteristics of women without human immunodeficiency virus (HIV) who acquired HIV infection during study.

	Mother #1	Mother #2	Mother #3	Mother #4
Study arm	Control	Control	Control	Intervention
Age at enrollment (years)	20	23	21	17
Marital Status	Married	Married	Never married, not living with partner	Never married, not living with partner
Education	Some secondary	Completed primary	Some secondary	Completed secondary
Partner age (years)	32	31	27	23
Reported partner HIV status	Negative for HIV at enrollment and 6 weeks (tested within last 3 months); no new test for partner reported at visits 14 weeks–24 months	Unknown status throughout participation in study	Unknown status at enrollment; reported new partner of unknown status at 12 months post-partum	Negative for HIV at enrollment (last tested > 3 months ago); no additional tests reported
Visit at first positive HIV test	14 weeks post-partum	12 months post-partum	12 months post-partum	18 months post-partum
Infant HIV test results	HIV-positive (14 weeks post-delivery)	HIV-negative	HIV-negative	HIV-negative

During the course of the study, twelve women died: three women with HIV during pregnancy and seven women with HIV and two women without HIV during the postpartum period (Figure 1).

### Child outcomes

There were 23 miscarriages prior to 28 weeks gestation - two among women without HIV and 21 among women with HIV. There were 26 stillbirths: six and 20 among women without and with HIV, respectively. During study follow-up, there were 33 deaths among infants born to mothers with HIV (18 intervention, 15 control), including one infant with HIV infection, and 12 deaths among infants born to mothers without HIV (8 intervention, 4 control).

Among infants born to mothers known to be living with HIV at enrollment, 9/358 (2.5%) infants were identified as having acquired HIV infection by 18 months postpartum (Table 5). There was no significant difference in HIV acquisition between study arms, with 5/166 (3.0%) and 4/191 (2.1%) infants acquiring HIV infection in the control and intervention arms, respectively. Nearly all (97.9%) infants born to mothers known to have HIV at enrollment were started on nevirapine (NVP) infant prophylaxis after delivery; of eleven infants born to women with HIV who did not receive NVP infant prophylaxis, two acquired HIV infection (18.2%). Eight of the nine mothers of infants who acquired HIV infection had first initiated ART during pregnancy. Four mothers of infants who acquired HIV (all in the control arm) had documented sub-optimal adherence to ART during pregnancy.

**TABLE 5:** Characteristics of children who acquired human immunodeficiency virus (HIV).

	Study arm	Infant age at visit at diagnosis	Maternal age (years)	EGA at 1^st^ ANC	Timing of maternal ART (EFV/3TC/TDF)	Maternal ART adherence and VL during pregnancy (if available)	Received infant NVP prophylaxis?
#1	Control	6 weeks	18	16	Started before pregnancy	Sub-optimal adherence	Yes, but missed more than once in a week (ran out of medicine)
#2	Control	6 weeks	22	–	Started at first ANC visit	Sub-optimal adherence	Yes
#3	Intervention	6 weeks	37	30	Started at first ANC visit	Adherence unknown	Yes
#4	Intervention	6 weeks	23	28	Started at first ANC visit	Good adherence	Yes
#5 (twin)	Intervention	6 weeks	31	20	Started at first ANC visit	Good adherence	No
#6	Intervention	6 weeks	26	20	Started at first ANC visit	Good adherence VL = 1,447 c/mL at time of delivery	Yes
#7	Control	14 weeks	20	24	Started at first ANC visit	Sub-optimal adherence VL = 26,694 c/mL during pregnancy	No
#8 (SC mother)	Control	14 weeks	20	26	NA- mother and infant tested positive at same visit (mother not retested prior to delivery)	NA	NA
#9	Control	12 months	27	31	Started at first ANC visit	Sub-optimal adherence	Yes
#10	Control	18 months	36	22	Started shortly after first ANC visit	Good adherence	Yes

ANC, antenatal care; ART, antiretroviral therapy; c/mL, copies/mL; EFV/3TC/TDF, efavirenz/lamivudine/ tenofovir disoproxil fumarate; EGA, estimated gestational age; NA, not available; NVP, nevirapine infant prophylaxis; SC, seroconverting mother; VL, viral load.

### Patient satisfaction

**FIGURE 2:**
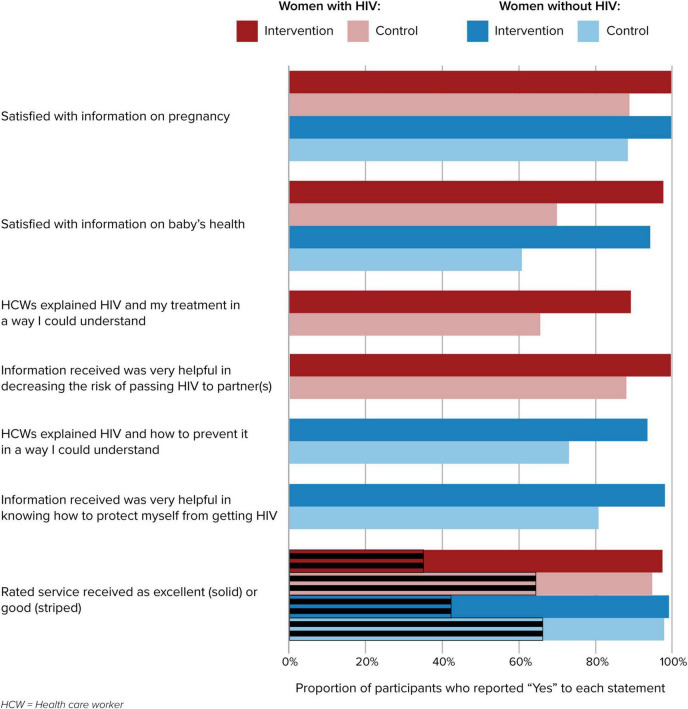
Participant satisfaction with information and services provided by facility personnel.

Patient satisfaction with services was assessed at each study visit. Women in the intervention arm, regardless of HIV status, reported significantly higher levels of satisfaction with the information and service provided during their clinic visits (*p* < 0.05); results are shown for the 12 months postpartum visit, though this trend was sustained over all study visits (Figure 2).

## Discussion

Implementation of a multidisciplinary facility-level intervention designed to coordinate patient-centered MCH and PVT services was effective in improving the quality of services provided to pregnant and postpartum women and some key PVT and MCH outcomes. Elimination of vertical HIV transmission will require sustained, effective services and support for both women living with and without HIV to address all four prongs of the WHO PVT strategy (1, 38). Women must also feel confident that the services in their communities will provide clear and accurate information, support for the specific challenges they face, and a non-stigmatizing environment (7, 39, 40). Improvements in the quality and coordination of care provided to individuals with HIV or at risk of contracting HIV will also help to facilitate the successful introduction of new biomedical innovations in HIV prevention and treatment.

Regarding primary prevention (prong 1), we found that women without HIV receiving the intervention reported higher satisfaction with the HIV prevention information they received and were significantly more likely to be retested for HIV during pregnancy. However, even in the intervention arm, nearly 25% of women did not have repeat testing prior to delivery or between delivery and 12 months postpartum despite national guidelines. The control arm mother who seroconverted after enrollment and had an infant who acquired HIV did not have a repeat HIV test around the time of delivery - a missed opportunity to intervene with enhanced infant antiretroviral prophylaxis had she been identified as infected with HIV prior to breastfeeding. Support for primary prevention among women without HIV is a critical gap in many PVT programs, particularly in areas with high HIV prevalence where sero-incident infections and lack of early identification to initiate PVT interventions drives many new infections in infants (1, 35, 41–43).

While PVT prong 2 is prevention of unintended pregnancy, prevalence of modern contraceptive use postpartum is low in sub-Saharan Africa, particularly among women with HIV, resulting in high rates of unplanned/unintended pregnancy and high unmet need for family planning (44–47). The IMPROVE intervention significantly increased consistent postpartum use of modern contraception among both women with and without HIV compared with the control arm. However, while significantly improved, less than half of postpartum women reported consistent contraception use even in a setting where family planning commodities were available.

Early identification of HIV infection and ART initiation in pregnant women with HIV, with support for consistent adherence to achieve viral suppression and improve maternal health, are critical to achieving the goals of WHO prong 3 (provision of maternal ART for PVT). Lesotho has made remarkable progress, as evidenced in our study by all women with known HIV receiving ART and an overall 18 months vertical transmission rate of 2.5%. However, maternal ART adherence and retention in care remains a challenge in Lesotho and other sub-Saharan African countries (47, 48). Women with HIV in IMPROVE intervention facilities were significantly more likely to consistently adhere to ART than women in the control arm, with a trend toward higher rates of undetectable VL at 12 months postpartum. Over half of the infants who acquired HIV infection in our study were born to mothers with documented sub-optimal adherence and/or viremia during pregnancy, consistent with reports from other studies by our team and other teams in the region (49–52). Despite universal ART use, we found higher maternal mortality among women with HIV, similar to a report on maternal deaths from cohorts in Malawi, Tanzania and South Africa, which found a 5-fold increased risk of maternal mortality in women living with HIV compared to women without HIV (53). We previously reported separately on increased adverse pregnancy outcomes among women with HIV compared to women without HIV in this cohort (54).

Evidence shows that just offering care and treatment is not sufficient to ensure healthy outcomes for women living with HIV and their children. In PVT prong 4, comprehensive support from peers, HCW, and CHW was utilized to help women navigate specific challenges, such as partner disclosure, stigma, gender-based violence, access to appropriate services and other issues. Through expanding community visits, providing job aides to HCW/CHW that ensured clear and consistent messaging from all levels of care providers, and enhancing PHDP positive counselling skills in those providing services to pregnant and postpartum women, the IMPROVE intervention sought to improve the support provided to both women with and without HIV. Several studies have reported that community support improves MCH outcomes, through CHW or peer support visits to pregnant women to reinforce counselling messages, respond to outstanding patient questions, follow-up late or missed visits and provide referrals to needed services (55–57).

One mechanism by which the IMPROVE intervention expected to improve outcomes was increasing the quality of services; minimizing the burden of attending visits; providing accessible and useful information on pregnancy, infant health, and HIV; and delivering services in a respectful and tailored manner. Our data indicate that the intervention was well-received; women in the intervention arm consistently reported significantly higher satisfaction with their clinic visits throughout the duration of the study. This may have also contributed to the higher proportion of women at intervention sites having the recommended 4 ANC visits and continuing to return to the sites for post-partum care.

One of the strengths of the IMPROVE intervention was the specificity of the facility-level changes made in response to the needs and situation of those facilities. While the study team provided a standard package of ongoing support and training to each intervention facility, each site had significant latitude in how to identify and address obstacles to deliver higher-quality, more efficient service to clients. However, this variation across facilities presents a challenge in identifying the effect of individual components of the intervention package and comparing the effectiveness of the intervention package overall, even after adjusting for clustering in the analyses.

A limitation of the study was reliance on data collected from or following routine clinical visits at study sites. Voluntary withdrawal from the study, transfer/relocation, loss to follow-up from routine care, all limited the ability of the study to evaluate outcomes at later time points with the desired precision. Among both women with and without HIV, it was not uncommon for women to relocate after the birth or to choose to receive services at a different location at some point during the course of the study (both elsewhere in Lesotho, or in neighboring South Africa). Documentation of postpartum retention was particularly challenging for women without HIV, who did not have routine clinic visits after 14 weeks postpartum, and sometimes chose to bring their infants to a mobile vaccination clinic rather than return to the facility. The study team made extensive efforts to determine and document whether a participant was receiving services at another health facility, particularly once it became clear from interim data review that there was a high rate of transfer and relocation. Similar studies should ensure that robust systems for tracking participants are in place from the beginning of the study, rather than relying primarily on standard procedures at resource-constrained local facilities. Discontinuation of data collection activities before all participants were able to complete the full 24 months postpartum period follow-up further limited our ability to assess outcomes at later time periods and determine whether positive changes were sustained over time.

## Conclusion

The IMPROVE interventions were designed to be feasible to implement in routine care settings with existing staff and minimal additional resources. As this intervention builds primarily on the current healthcare system and infrastructure, scaling up the intervention should not require a significant amount of additional financial or human resources. Overall, implementation of the IMPROVE interventions was found to be an effective strategy to enhance MCH/PVT service delivery and improve provider-patient interaction.

## Data Availability

The data that support the findings of this study are available from the Elizabeth Glaser Pediatric AIDS Foundation, Lesotho but restrictions apply to the availability of these data, which were used for the current study, and so are not publicly available. Data are however available from the authors upon reasonable request and with permission of the Lesotho Ministry of Health.
